# Application of Artificial Intelligence in Neuroendocrine Lung Cancer Diagnosis and Treatment: A Systematic Review

**DOI:** 10.7759/cureus.61012

**Published:** 2024-05-24

**Authors:** Sindhu C Pokhriyal, Abhishek Shukla, Uma Gupta, Muthanna Mohammed Hasan Al-Ghuraibawi, Ruchi Yadav, Kalpana Panigrahi

**Affiliations:** 1 Internal Medicine, One Brooklyn Health - Interfaith Medical Center, Brooklyn, USA; 2 School of Information Studies, Syracuse University, Syracuse, USA; 3 Hematology and Oncology, Brookdale University Hospital Medical Center, Brooklyn, USA

**Keywords:** diagnosis, lung, intelligence, artificial, neuroendocrine

## Abstract

Neuroendocrine tumors (NETs) represent a heterogeneous group of neoplasms with diverse clinical presentations and prognoses. Accurate and timely diagnosis of these tumors is crucial for appropriate management and improved patient outcomes. In recent years, exciting advancements in artificial intelligence (AI) technologies have been revolutionizing medical diagnostics, particularly in the realm of detecting and characterizing pulmonary NETs, offering promising avenues for improved patient care.

This article aims to provide a comprehensive overview of the role of AI in diagnosing lung NETs. We discuss the current challenges associated with conventional diagnostic approaches, including histopathological examination and imaging modalities. Despite advancements in these techniques, accurate diagnosis remains challenging due to the overlapping features with other pulmonary lesions and the subjective interpretation of imaging findings.

AI-based approaches, including machine learning and deep learning algorithms, have demonstrated remarkable potential in addressing these challenges. By leveraging large datasets of radiological images, histopathological samples, and clinical data, AI models can extract complex patterns and features that may not be readily discernible to human observers. Moreover, AI algorithms can continuously learn and improve from new data, leading to enhanced diagnostic accuracy and efficiency over time.

Specific AI applications in the diagnosis of lung NETs include computer-aided detection and classification of pulmonary nodules on CT scans, quantitative analysis of PET imaging for tumor characterization, and integration of multi-modal data for comprehensive diagnostic assessments. These AI-driven tools hold promise for facilitating early detection, risk stratification, and personalized treatment planning in patients with lung NETs.

## Introduction and background

Lung cancer is a significant global health issue, being the leading cause of cancer-related deaths worldwide. In 2020, the USA reported the second-largest number of lung cancer cases worldwide. Approximately, 229,000 new cases, accounting for 12.7% of all cancer diagnoses, with an incidence rate of 45.6/100,000 were reported in the USA [1-3]. In the USA, lung cancer is the second most common cancer in both men and women, with about 234,580 new cases and 125,070 deaths estimated for 2024 [4]. The lifetime chance of developing lung cancer is about 1 in 16 for men and 1 in 17 for women [4,5]. Despite being a leading cause of cancer death, the number of new cases and deaths from lung cancer has been decreasing due to factors like smoking cessation and advancements in early detection and treatment [4,5].

Lung neuroendocrine tumors, also known as LNETs, are tumors that originate in amine precursor uptake and decarboxylation (APUD) neuroendocrine cells. Some researchers believe that LNETs account for 20% of the total number of lung cancer cases [6]. These tumors are extremely rare and have a heterogeneous malignancy and are typically asymptomatic and non-functional [7]. When it comes to the localization of NETs, the lung is the second organ after the liver. Lung NETs account for 25% of all NETs and anywhere from 1% to 2% of all cancers [8]. When it comes to diagnosis and treatment, combined types of LNETs continue to be a challenge, with diagnosis often being incidental and time-consuming, and treatment being focused on functional tumors [9].

AI has significantly impacted lung cancer diagnosis and treatment. Studies show that AI-assisted diagnostic systems offer high diagnostic value for lung cancer, improving efficiency and accuracy in diagnosis. AI applications in lung cancer screening have aided in early identification of cancerous lung nodules, enabling timely treatment initiation, and improving patient outcomes [10]. High-accuracy AI algorithms in radiomics have also been instrumental in enhancing radiologist performance in detecting lung cancers on chest X-rays, leading to improved detection performance. The integration of AI into lung cancer care shows great promise for enhancing early detection, treatment planning, and patient outcomes [10-12]. The current review is designed with the purpose of studying the advancements in the role of AI in diagnosing as well as managing lung NETs.

## Review

Methodology

The Preferred Reporting Items for Systematic Reviews and Meta-Analyses (PRISMA) 2020 criteria served as the basis for the systematic review that was carried out.

Search strategy & eligibility criteria

The study comprised searching for relevant literature on the role of AI in fine-tuning diagnosis, workup, and treatment of lung NETs. We researched databases like PubMed, PubMed Central (PMC), Google Scholar, and Clinicaltrials.gov. Exclusion criteria included studies on those less than 18 years of age, and excluding articles related to neuroendocrine tumors of the gastrointestinal tract. All studies included in the study had full text available for review and analysis.

We used various combinations of our keyword concepts, including artificial intelligence, lung, neuroendocrine tumors, and diagnosis to search all databases. PubMed's Medical Subject Headings (MeSH) database was used for refining the search strategy further (as depicted in Table 1).

**Table 1:** Keywords employed in the study. MeSH: medical subject headings

Search Strategy	Keywords
Regular keywords	Artificial, Intelligence, AI, Lung, neuroendocrine, diagnosis
MeSH keywords	("artificial intelligence"[MeSH Terms] OR ("artificial"[All Fields] AND "intelligence"[All Fields]) OR "artificial intelligence"[All Fields]) AND ("lung"[MeSH Terms] OR "lung"[All Fields]) AND ("neurosecretory systems"[MeSH Terms] OR ("neurosecretory"[All Fields] AND "systems"[All Fields]) OR "neurosecretory systems"[All Fields] OR "neuroendocrine"[All Fields]) AND ("diagnosis"[Subheading] OR "diagnosis"[All Fields] OR "diagnosis"[MeSH Terms])

Selection process & quality appraisal

**Figure 1:**
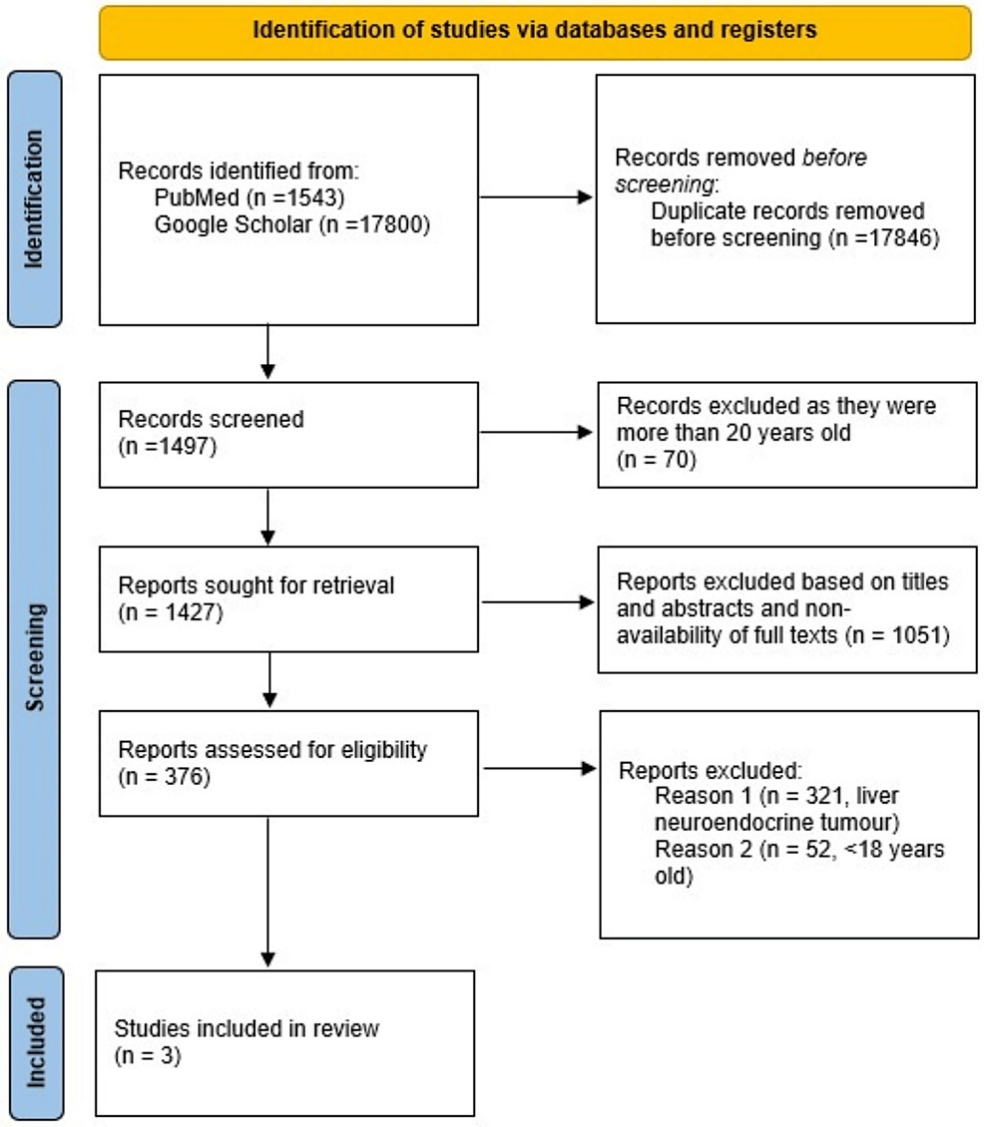
PRISMA 2020 flow diagram for new systematic reviews which included searches of databases and registers

The articles included in the final review were randomized controlled trials, retrospective observational studies, meta-analyses, cohort studies, and systematic reviews on artificial intelligence in lung adenocarcinoma (as depicted in Figure 1). The articles that were chosen from all of the databases were reviewed by four different reviewers independently in order to guarantee a thorough analysis of the existing body of research on artificial intelligence in improving the diagnosis and workup of lung neuroendocrine tumors.

Newcastle Ottawa Quality Matrix, which is a critical appraisal tool for systematic reviews, was utilized in order to incorporate publications that were pertinent to the subject matter of the role of artificial intelligence in diagnosing and managing lung NETs (as shown in Table 2). We identified three articles that studied the use of artificial intelligence in the diagnosis and workup of lung neuroendocrine tumors. 

**Table 2:** Newcastle Ottawa Quality Matrix for quality appraisal of systematic review

NOS Items	Ilie et al. [13]	Alcala et al. [14]	Carlsen et al. [15]
Representativeness of the exposed cohort	0	0	1
Selection of the non-exposed cohort	0	0	1
Ascertainment of exposure	1	1	1
Demonstration that outcome of interest was not present at the start of the study	1	0	1
Comparability of cohorts on the basis of the design and analysis	1	0	1
Assessment of outcome	1	1	1
Was follow-up long enough for outcomes to occur	1	0	0
Adequacy of follow-up of cohorts	0	0	0
Total	5	2	6

Discussion

The average time to diagnose a rare disease like a lung NET can be as long as seven years [16]. Most of the LNETs are accidentally discovered during imaging done for other medical indications [17]. Lung NETs are at present histologically graded into typical carcinoids, atypical carcinoids, large cell neuroendocrine tumors (LCNEC), and small cell lung cancers (SCLC). Typical carcinoids are low-grade malignant tumors that tend to have a good prognosis for survival. Atypical carcinoids are intermediate-grade malignant tumors that behave more aggressively in the body. LCNEC and SCLC are high-grade malignant tumors that are associated with a poor prognosis [18]. The Ki-67 antigen labeling index, which is an indicator of proliferating tumor cells, is not considered standard in the diagnosis or grading of lung NETs [19]. Gene rearrangements in lung neuroendocrine tumors (NETs) can be identified using a variety of techniques, including next-generation sequencing (NGS), fluorescence in situ hybridization (FISH), and immunohistochemistry (IHC). These methods are preferred for the diagnosis, grading, and prognostication of lung NETs due to their precision and reliability. NGS techniques have the remarkable ability to process billions of DNA strands in a shorter amount of time while laying the groundwork for precision medicine [20,21].

Redemann et al. investigated if a deep-learning convolutional neural network (CNN) could outperform existing IHC profiles in accurately identifying the point of origin of the NET [21]. Despite the fact that IHC successfully recognized 76% of these cases and CNN accurately detected 70%, these results failed to show statistical significance [21]. Since the study was conducted in 2020, several CNN stability training exercises have claimed to improve the robustness of CNN to scanner and IHC-based image variability, without the need for more data [22]. They analyzed the potential of deep learning approaches in appropriately classifying the tumor microenvironment in these patients. Although his study was generalized to all lung cancer patients IHC used in lung neuroendocrine tumors includes synaptophysin, chromogranin, CD56, TTF-1, and Ki67 labeling index which have been useful in differentiating between typical carcinoids, atypical and small cell carcinomas in the lung [23]. Wang et al. in their study created an IHC-based model in lung cancer patients which was based on freely available IHC data that had been already annotated by pathologists [24].

Mitotic counts per 2 mm^2^ are the standard histological criteria used to distinguish between typical carcinoid, atypical carcinoid, and neuroendocrine cancer. The presence of necrosis is an additional factor for atypical carcinoids. About 70% of the diagnostic samples are either cytological samples or crushed biopsies, which are usually not well preserved morphologically, making morphological and IHC examination challenging. Even amongst skilled thoracic pathologists, there are borderline neuroendocrine neoplasms that morphologically lie between the several histotypes, and can lead to ambiguity. To help pathologists differentiate histologic subtypes and classify lung neuroendocrine neoplasms, Ilié et al. used a deep learning classifier fitted with a convolutional neural network (CNN) [13]. According to their study, histopathological whole-slide images can be utilized to aid in the detection of pulmonary NETs using CNNs. This is the first study that we are aware of which looks into the use of CNNs to distinguish between various pulmonary NET subtypes and they trained three CNN algorithms to identify the different subtypes of LNETs. Even in complicated cases, like mixed SCLC with LCNEC, the CNNs were able to reach accuracy comparable to pathologists, expanding precision medicine's use and efficiency. This means that pathologists may rely on morphology as described and diagnosed by the trained AI tool when examining tumor tissue and may only require immunostaining in the most challenging cases [13].

Alcala et al. evaluated and contrasted the molecular profiles of different lung NETs by combining transcriptome and methylome data and employing machine-learning (ML) and multi-omics factor analyses. The machine learning classifier developed by them was based on genome-wide expression or methylation data and was unable to distinguish between atypical and typical carcinoids: 64-83% of typical carcinoids were correctly categorized, whereas only 30-41% were correctly classified as atypical carcinoids. The differential expression study revealed that atypical carcinoids have very few core differentially expressed genes and different methylation sites. Overall, these findings indicated that the histological categorization does not fully correspond to the molecular classification [14].

In the study by Carlsen et al., patients with lung neuroendocrine neoplasms who had [64Cu] Cu-DOTATATE PET/CT were included in the study and their most significant conclusion was that using a convolutional neural network reduced the time spent on the tedious task of total tumor segmentation in patients with neuroendocrine neoplasms from 20 to 5 minutes. AI segmentations were applied in a clinical imaging viewer, and a physician evaluated performance and made manual modifications [15].

There are new findings that indicate that typical and atypical carcinoids are primarily altered in genes that are responsible for chromatin remodeling. On the other hand, LCNEC and SCLC are also mutated in genes that regulate cell differentiation and cell cycle checkpoints. Thus, a new grading system in lung NETs that serves as a bridge connecting molecular alterations, morphological characteristics, and clinical behavior could provide a better stratification of prognostic classes [20].

Various models and types of CNN can be employed to simplify and assist in various aspects of the diagnosis of lung NET. CNN can be used in characterizing suspicious lung nodules on imaging, in simplifying tumor segmentation, in whole slide imaging, IHC, and perhaps even in NGS. We suggest a comprehensive data fusion approach enhances feature representation, classification performance, interpretability, and personalized medicine potential, driving advancements in cancer research and clinical practice.

Limitations

This review yielded a small number of studies, and all the studies were heterogeneous in the parameters that were studied. Although each study discussed the application of AI in the diagnosis and workup of lung NETs they covered different aspects of the workup.

## Conclusions

While the promise of AI in diagnosing lung NETs is undeniable, it's crucial to acknowledge that its application in this specific domain is still in its nascent stages. Despite significant advancements in AI algorithms and computational capabilities, the development and validation of AI-based tools tailored specifically for lung NET diagnosis are relatively limited. Current AI models frequently depend on datasets that have inherent biases or lack diversity. These datasets are often difficult to access and are not standardized. This may compromise their generalizability and robustness in real-world clinical settings. Thus, several challenges must be addressed to realize the full potential of AI in clinical practice, including the need for standardized datasets, robust validation studies, regulatory approval, and integration into existing workflows. Ethical considerations, such as patient privacy and transparency in algorithmic decision-making, also warrant careful attention. In conclusion, collaborative efforts between clinicians, researchers, and industry stakeholders are essential to harnessing the full potential of AI in this critical area of pulmonary oncology.

## References

[1] Thandra KC, Barsouk A, Saginala K, Aluru JS, Barsouk A (2021). Epidemiology of lung cancer. Contemp Oncol (Pozn).

[2] (2023). Lung cancer - World Health Organization. https://www.who.int/news-room/fact-sheets/detail/lung-cancer.

[3] Li C, Lei S, Ding L (2023). Global burden and trends of lung cancer incidence and mortality. Chin Med J (Engl).

[4] (2024). Lung Cancer Statistics | How Common is Lung Cancer?. https://www.cancer.org/cancer/types/lung-cancer/about/key-statistics.html.

[5] Jani CT, Singh H, Abdallah N (2023). Trends in lung cancer incidence and mortality (1990-2019) in the United States: a comprehensive analysis of gender and state-level disparities. JCO Glob Oncol.

[6] Benzerdjeb N, Berna P, Sevestre H (2017). GLUT1: A novel tool reflecting proliferative activity of lung neuroendocrine tumors?. Pathol Int.

[7] Wolin EM (2017). Advances in the diagnosis and management of well-differentiated and intermediate-differentiated neuroendocrine tumors of the lung. Chest.

[8] Yao JC, Hassan M, Phan A (2008). One hundred years after "carcinoid": epidemiology of and prognostic factors for neuroendocrine tumors in 35,825 cases in the United States. J Clin Oncol.

[9] Savu C, Melinte A, Diaconu C (2022). Lung neuroendocrine tumors: a systematic literature review (Review). Exp Ther Med.

[10] Liu M, Wu J, Wang N (2023). The value of artificial intelligence in the diagnosis of lung cancer: a systematic review and meta-analysis. PLoS One.

[11] Cellina M, Cacioppa LM, Cè M (2023). Artificial intelligence in lung cancer screening: the future is now. Cancers (Basel).

[12] Gao Q, Yang L, Lu M, Jin R, Ye H, Ma T (2023). The artificial intelligence and machine learning in lung cancer immunotherapy. J Hematol Oncol.

[13] Ilié M, Benzaquen J, Tourniaire P (2022). Deep learning facilitates distinguishing histologic subtypes of pulmonary neuroendocrine tumors on digital whole-slide images. Cancers (Basel).

[14] Alcala N, Leblay N, Gabriel AA (2019). Integrative and comparative genomic analyses identify clinically relevant pulmonary carcinoid groups and unveil the supra-carcinoids. Nat Commun.

[15] Carlsen EA, Lindholm K, Hindsholm A (2022). A convolutional neural network for total tumor segmentation in [(64)Cu]Cu-DOTATATE PET/CT of patients with neuroendocrine neoplasms. EJNMMI Res.

[16] Hasani N, Farhadi F, Morris MA (2022). Artificial intelligence in medical imaging and its impact on the rare disease community: threats, challenges and opportunities. PET Clin.

[17] Jeung MY, Gasser B, Gangi A (2002). Bronchial carcinoid tumors of the thorax: spectrum of radiologic findings. Radiographics.

[18] Travis WD, Brambilla E, Nicholson AG (2015). The 2015 World Health Organization classification of lung tumors: impact of genetic, clinical and radiologic advances since the 2004 classification. J Thorac Oncol.

[19] Pelosi G, Papotti M, Rindi G, Scarpa A (2014). Unraveling tumor grading and genomic landscape in lung neuroendocrine tumors. Endocr Pathol.

[20] Simbolo M, Mafficini A, Sikora KO (2017). Lung neuroendocrine tumours: deep sequencing of the four World Health Organization histotypes reveals chromatin-remodelling genes as major players and a prognostic role for TERT, RB1, MEN1 and KMT2D. J Pathol.

[21] Redemann J, Schultz FA, Martinez C, Harrell M, Clark DP, Martin DR, Hanson JA (2020). Comparing deep learning and immunohistochemistry in determining the site of origin for well-differentiated neuroendocrine tumors. J Pathol Inform.

[22] Miranda Ruiz F, Lahrmann B, Bartels L (2023). CNN stability training improves robustness to scanner and IHC-based image variability for epithelium segmentation in cervical histology. Front Med (Lausanne).

[23] Pasala UJ, Hui M, Uppin SG, Kumar NN, Bhaskar K, Paramjyothi GK (2021). Clinicopathological and immunohistochemical study of pulmonary neuroendocrine tumors - A single-institute experience. Lung India.

[24] Wang R, Qiu Y, Wang T (2024). MIHIC: a multiplex IHC histopathological image classification dataset for lung cancer immune microenvironment quantification. Front Immunol.

